# Effect of Physical Guidance on Learning a Tracking Task in Children with Cerebral Palsy

**DOI:** 10.3390/ijerph18137136

**Published:** 2021-07-03

**Authors:** Hadi Nobari, Elham Azimzadeh, Hamidollah Hassanlouei, Georgian Badicu, Jorge Pérez-Gómez, Luca Paolo Ardigò

**Affiliations:** 1 Department of Physical Education and Sports, University of Granada, 18010 Granada, Spain; 2 HEME Research Group, Faculty of Sport Sciences, University of Extremadura, 10003 Caceres, Spain; jorgepg100@gmail.com; 3 Department of Behavioural, Cognitive and Technology Sciences in Sport, Faculty of Sport Sciences and Health, Shahid Beheshti University, Tehran 198396-3113, Iran; Hamidhasanlooie@gmail.com; 4 Department of Physical Education and Special Motricity, Transilvania University of Brasov, 500068 Brasov, Romania; georgian.badicu@unitbv.ro; 5 Department of Neurosciences, Biomedicine and Movement Sciences, School of Exercise and Sport Science, University of Verona, 37131 Verona, Italy; luca.ardigo@univr.it

**Keywords:** physical guidance, tracking task, cerebral palsy, challenge point framework, frequency

## Abstract

The purpose of this study was to investigate the effect of physical guidance (PG) frequency on learning a tracking task in children with hemiplegic spastic cerebral palsy (CP). For this purpose, 25 children, aged 7–15 years with CP affecting the left side of the body, who were classified in levels II–III of Manual Abilities Classification System (MACS) and levels III–IV of Gross Motor Function Classification System (GMFCS), were recruited from 10 clinical centers. A pre-test including two blocks of 12 trials of the tracking task without any PG was performed by all participants, after that they were assigned into five homogenous groups (with 100%, 75%, 50%, 25%, and 0% of PG) through blocked randomization according to their age. All participants involved in an intervention consisted of eight sessions (four blocks of 12 trials in each session) practicing a tracking task. The 0% PG group received no PG, the 25% PG group received PG for three trials, the 50% PG group received PG for six trials, the 75% PG group received PG for nine trials, and the 100% PG group received PG for all twelve trials. PG consisted of placing the experimenter’s hand around the child’s less-involved hand guiding to stay on the track and complete the task. Learning was inferred by acquisition and delayed retention tests. The results showed that the higher frequency of PG led to more accurate performance during practice phase. However, the group that received 75% PG had significantly better performance compared to the other groups in the retention phase. It is concluded that optimum level of PG, about 75% of trials, can be helpful for learning a tracking task in children with spastic hemiplegic CP, supporting the challenge point framework.

## 1. Introduction

Children with cerebral palsy (CP) have weak physical abilities [1]. Approximately 2–2.5/1000 children have CP with affected muscle tone, limited range of movement and motor skills, often accompanied by intellectual, communication, and behavioral impairments. These limitations may have effect on performing everyday motor activities such as putting on clothes, eating, and walking; these children also engage in less physical activity compared to their typically developing peers [2,3]. Therapeutic interventions for children with CP targeting development of gross and fine motor skills, especially upper-limb functions and eye-hand coordination, improved their functional skills and encourage them to participate in social activities. One of the most important and common debilitating factors in CP is the malfunction in the upper extremities to perform motor skills and there is a strong evidence that task-specific training may lead to improve general upper limb function among this population [3].

In the field of motor learning, typically developing children are constantly learning new motor tasks during development. However, children with CP may use information in different way for learning motor skills depending on their stages of cognitive, sensory, and motor development [4]. The sensorimotor impairment is an important handicap in this disease which significantly impacts the quality of life in this population [5].

It has been reported that the sensorimotor impairments, visual perception, and motor planning deficits may affect motor learning in this population [6]. Previous studies showed that motor ability and performance in children with CP could be improved with physical practice [2,7].

Children with CP demonstrated more error than children with typical development in terms of accuracy and consistency during the acquisition, retention, and reacquisition phases, suggesting motor execution difficulties. These children often demonstrate different motor learning strategies due to sensory, motor execution, and cognitive impairments [8,9,10,11].

It is well documented that augmented feedback enhances acquisition and learning a motor task but there is little information available to guide practitioners in the effective use of feedback schedules to enhance acquisition and retention of motor skills in children with CP. A previous study examined the effect of augmented feedback on learning a new motor skill in adults with and without unilateral brain damage and showed that adults with unilateral brain damage exhibited more error than control participants as they practiced a rapid spatially and temporally constrained task (using their less-involved upper extremity). Differences between the groups were attributed to motor control and execution, not the cognitive learning of the motor skill [8].

One of the factors influencing learning motor skills is physical guidance (PG) that is recurrently used in education and rehabilitation. In this method, the learner is provided physical assistance during practice to facilitate the acquisition of the new skill and often involves recurrent direct guidance of the learner’s moving limbs (e.g., by pulling and pushing) [12]. It has been established that PG can play a key role in acquiring a motor skill [13]. However, it has been argued that guidance must be assistive rather than limiting [14]. Often, the patient with a sensorimotor disorder, such as CP, cannot recognize the movement errors with only the therapist’s verbal description, but through PG could be able to understand the spatial and temporal movement pattern and identify his/her own performance errors. Furthermore, throughout this process, the child with CP could possibly attend to appropriate wrist position, target accuracy, movement speed, and/or spatial trajectory simultaneously in a tracking task which performed by hands [15].

In order to help patients learn a skill, therapists initially provide PG, but ultimately, the primary goal is to assist the individual to develop the ability to control the movement on their own. It has been reported that PG effects in the retention phase [16]. A meta-analysis of 40 studies on learning of complex motor tasks in adults showed that continuous feedback may improve motor performance during the acquisition, but the faded feedback was more effective for the retention phase of learning [17]. Therefore, it is important to establish an optimal frequency of guidance, particularly PG, which is important for motor task learning.

According to the guidance hypothesis, practice with a high relative frequency of PG would be detrimental for learning. Although the guiding properties are beneficial for motor learning when used to reduce error, but detrimental when relied upon [18,19]. A previous study that investigated the feedback frequency in children with CP showed that continuous feedback (100%) compared to reduced (50%) or no (0%) feedback improved throwing accuracy in acquisition phase. However, only the reduced feedback group showed better performance in retention phase [2]. These results supported the guidance hypothesis [19]. However, another study indicated that the children with typical development, with 100% feedback had fewer errors than the 62% feedback group during acquisition and retention. However, there was no difference between feedback subgroups of children with CP [8].

Furthermore, according to the Challenge Point Framework (CPF), there is an interaction of the information processing capabilities of the learner, the task demands, and practice conditions which influence motor learning. Practice conditions may alter the difficulty e.g., the reduced frequency of PG could be more challenging for this population to learn a new motor task [8]. Although numerous studies have considered the effect of PG, we are not aware of any research examining the effects of different frequencies of PG on learning a motor task in children with CP, and furthermore, there is little specific evidence to guide the occupational therapists to implement the more effective frequencies of PG for intervention in this population [20]. Thus, the purpose of the present study was to investigate the predictions of the challenge point hypothesis for learning a novice motor task in children with spastic hemiplegic CP, which states that too much or too little information will retard learning by manipulating the frequency of PG (i.e., 25%, 50%, 75%, or 100%).

## 2. Materials and Methods

### 2.1. Participants

Twenty-five boys with hemiplegic spastic CP, aged from 7 to 15 years, who were classified in levels II–III of Manual Abilities Classification System (MACS) and levels III–IV of Gross Motor Function Classification System (GMFCS), were recruited from ten clinical centers. Inclusion criteria for all children were (1) no severe visual deficits, (2) classifying in levels II–III of MACS and levels III–IV of GMFCS, (3) hemiplegic CP affecting the left side of the body, and (4) the less-involved (preferred) hand was the right hand. Exclusion criteria were (1) additional orthopedic or neurological disorders, (2) disability in sitting independently, and (3) pharmacological or surgical procedures in the past year. Signed informed consent was obtained from all the parents. All participants were assigned into five homogenous groups (100%, 75%, 50%, 25%, and 0% of PG) through blocked randomization according to their age.

**Figure 1:**
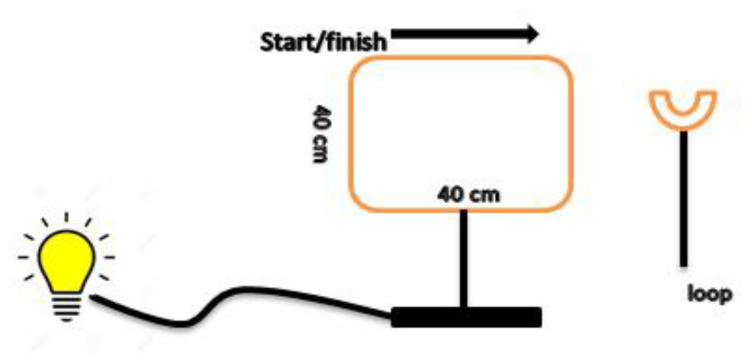
The apparatus for training the tracking task included a wire square trajectory which had 40 cm for each side and a light bulb that would turn on when the loop touched the wire. To accomplish one trial of the task, participants had to put the loop on the top and the left corner of the square and move the loop around the wire square to reach the start place again.

Participants sat in front of a table while grasping a loop with their less-involved (the right) hand to perform a tracking task. The task consisted of a wire square trajectory which had 40 cm for each side, like a wire loop game. The apparatus was sensitive to touch and there was a light bulb that would turn on when the loop touched the wire. In order to complete one trial of the task, participants put the loop on the top and the left corner of the square, and after hearing the “go” signal by the trainer, they moved the loop around the wire square to reach the start place again (Figure 1).

### 2.2. Sample Size

We evaluated power and sample size for the design based on the statistical approach analyzed—a priori: compute required sample size; F tests; one-way, fixed effects, omnibus; number of groups = 5; power (1-β err prob) = 0.60—based on a prior study on the effect of physical guidance frequency and motor learning in children with hemiplegic cerebral palsy, which found a large effect size [21]. With a total of 25 subjects, there is a 62.8% (actual power) chance of accurately rejecting the null hypothesis of no difference in variables. G-Power software was utilized for this statistical analysis (University of Dusseldorf, Dusseldorf, Germany) [22].

### 2.3. Procedure and Design

**Figure 2:**
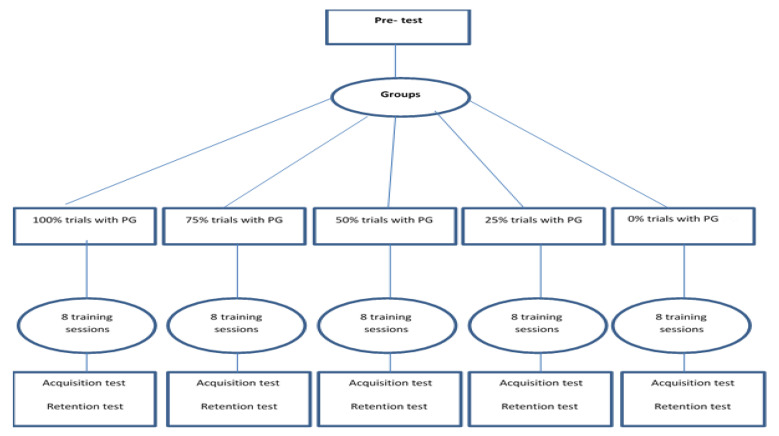
Research design.

This study was an experimental design with pre-test, acquisition, and retention tests (see Figure 2). In the first session, the participants learned how to perform the task. After demonstration of the skill, a pre-test involving two blocks of 12 trials of the tracking task without any PG was performed by all participants. They were asked to make the movement as accurate as possible (i.e., fewer errors). In the acquisition phase, all participants practiced the tracking task, which consisted of eight sessions every other day (four blocks of 12 trials in each session). The participants had 10 minutes’ rest after each block. PG was implemented by placing the trainer’s hand around the child’s less-involved hand (which was the right hand) and physically guiding it to complete the trial with the least number of errors. At each session, the group with 100% PG received PG in all twelve trials; the group with 75% PG received PG in nine trials (the first three trials out of every four trials); the group with 50% PG received PG in six trials (the first trial out of every two trials); the group with 25% PG received PG in three trials (the first trial out of every four trials); and the no-PG group received no PG (0% PG) in all twelve trials. All practice sessions were conducted in the same manner. An acquisition test was performed in the last session immediately after the trials were completed, and a delayed retention test was performed four days later. The same as the pre-test, the number of total touches (errors) in 24 trials was calculated as the scores of the tests.

### 2.4. Statistical Analysis

Shapiro–Wilk tests showed a normal distribution in all data (*p* > 0.05) and Levene’s test indicated homogeneity of variances between groups (*p* > 0.05) in the pre-test, acquisition, and retention phases. One-way ANOVA and Tukey’s HSD post-hoc were used to analyze acquisition and retention tests. The partial eta squared (ƞ^2^) was used to evaluate the effect size of the one-way ANOVA. Data are reported as means ± SD in the text and displayed as mean ± SE in the figures and table. A significance level of (*p* < 0.05) was used and all the analyses were performed with IBM Statistical Package for Social Sciences (SPSS, v25.0; IBM SPSS Inc., Chicago, IL, USA). An excel file was also used to draw the figures.

## 3. Results

General characteristics of the participants and descriptive statistics of dependent variables are shown in Table 1.

Table 2 illustrates the error values (mean ± SD) obtained by each group in all tests (i.e., pre-acquisition and retention tests).

**Figure 3:**
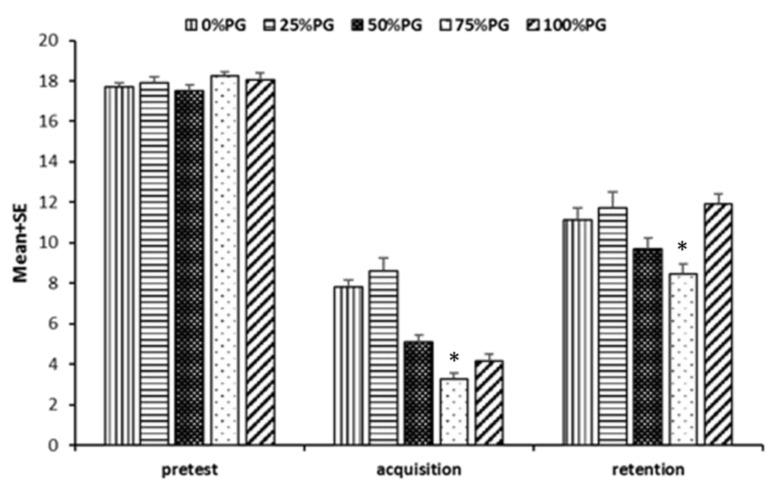
Performance of the groups in the tracking task tests. * Represents a statistically significant difference compared to the 75% PG group with the other groups (i.e., 0%, 25%, and 50% PG groups).

The results of one-way ANOVA showed that there were no significant differences between groups in the pretest (F_4, 20_ = 1.00, *p* = 0.43, ƞ^2^ = 0.17). However, in the acquisition test, there was a significant difference between groups (F_4, 20_ = 32.73, *p* = 0.001, ƞ^2^ = 0.87). Tukey’s HSD post-hoc test showed that the 75% PG group (fewer errors) had significantly better performances than 0%, 25%, and 50% PG groups (Figure 3); 100% PG was better than 0% PG and 25% PG; and the 50% PG group performed better than the 0% and 25% groups. There were no significant differences between other groups. In the retention test, one-way ANOVA was significant (F_4, 20_ = 6.15, *p* = 0.002, ƞ^2^ = 0.55). Therefore, according to Tukey’s HSD post-hoc test, the 75% PG group had significantly better performance than the 0%, 25%, and 100% PG groups, but there were no significant differences between other groups (Table 3).

## 4. Discussion

Our study investigated the effect of different frequencies of PG in learning a tracking task in children with hemiplegic CP. The results showed that the participants who received 75% PG had significantly better performance compared to the 0%, 25%, and 50% PG groups; and the 100% PG group performed better than the 0% and 25% PG groups in the acquisition test. In the retention test, the 75% PG group had significantly fewer errors in comparison to the 0%, 25%, and 100% PG groups; however, there were no significant differences between other groups (Figure 2).

The findings were consistent with previous studies with respect to the benefits of PG, which is a simple and non-invasive method for motor learning improvement. It can also help the child to selectively attend to the model’s erect posture and fluid wrist movement while reaching for the target in upper-extremity control [15]. This is more critical for children with sensorimotor dysfunction such as CP, so it may be useful for detecting the correct position of the limbs, which was the upper limb and specifically the wrist in the present study. Moreover, according to Bernstein (1967), exploration of the relationship between a movement and the physical environment is necessary for learning motor skills, and a set of movement solutions based on the large number of joint rotations (i.e., kinematic degrees of freedom) is needed to complete the desired movement. PG likely facilitates this process [6].

Furthermore, the results of the present study confirm the guidance hypothesis and the CPF, which state that too much or too little information will retard learning a new motor skill both in children with spastic hemiplegic CP and typically developing children. Moreover, our results support the findings of [12,16,23], confirming the beneficial effect of optimal frequency of PG on learning motor skills. Furthermore, in the study of Hemayattalab, et al. [2], only the group that received reduced feedback improved accuracy in throwing in the retention phase—this was in line with our findings. One explanation for these findings is that a high level of PG (i.e., 100%) makes the individual more dependent and therefore not able to utilize his/her internal sensory information. A low frequency of guidance (i.e., 25% or 0%), on the other hand, is not helpful in identifying and developing appropriate patterns of motor skills. In fact, PG should be provided at an optimum level to decrease the child’s dependency and to help them to identify appropriate patterns of skill. Continuous feedback limits the opportunity for exploration the instructions and information on each trial and may induce dependency in learner. In other words, reduced feedback or information results in a self-regulatory strategy in learning motor skills [8]. Therefore, an increased efficiency in encoding process and improved performance in the retention phase is expected [24].

Our results indicated that the optimal frequency of PG played a critical role in learning a tracking task in children with CP and likely this population may benefit from an optimal level of PG to get the appropriate amount of information as typically developing children confirming the CPF [25]. Therefore, an optimum level of PG for the children with CP would provide two important sets of resources for learning motor skill simultaneously. In this way, these children are able to identify appropriate patterns of motor skill using extrinsic information they receive through PG. Furthermore, implementing an appropriate level of PG may improve learning by activating the intrinsic feedback mechanisms [24].

Moreover, interventions involving task specificity in children with CP often relates to training of upper limb or fine motor activities. Task-specific training (TST) involves principles of motor learning with components including context, practice, and frequency of feedback. TST should involve varied components depending on the requirements of the skill, the environment, and the function of the child [3].

### Limitations

There were some limitations in the present study that need to be considered in future research. First is the small sample size and the ability to generalize the results to all children with hemiplegic CP. In addition, this is stated in the statistical power reported in the article method with 62%, so great caution should be taken when interpreting the results of the study and making conclusions. Second is the lack of control for additional variables influencing learning a motor task in CP children such as their cognitive abilities. Third is the comparison of the intervention on the affected hand and the less-involved one due to the application of therapeutic interventions. For this reason, we strongly recommend researchers interested in the neurophysiology of motor training in CP children to practice and experiments with the injured arm in studies to increase performance in hemiplegia, because the quality of life of these patients depends strongly on the residual capacities of the affected arm.

## 5. Conclusions

In summary, our results suggest that children with spastic hemiplegic CP had benefited from an optimal amount of PG (about 75% of trials) when learning a new skill with their less-involved hand. The results of this study may help physiotherapists to provide effective therapeutic interventions to improve motor learning in children with CP [26].

## Figures and Tables

**Table 1:** General characteristics of the participants by physical guidance.

Characteristics	Groups of PG										Totally (*n* = 5)	
	0% (*n* = 5)		25% (*n* = 5)		50% (*n* = 5)		75% (*n* = 5)		100% (*n* = 5)			
	Mean	SD	Mean	SD	Mean	SD	Mean	SD	Mean	SD	Mean	SD
Age (years)	11.00	1.581	11.00	2.55	11.20	2.95	11.20	2.95	11.00	2.236	11.08	2.29
Height (cm)	133.2	8.468	133.80	13.9	133.8	15.9	133.0	14.5	132.6	11.69	133.2	12.04
Body mass (kg)	32.00	5.24	32.40	8.64	33.00	9.82	33.60	9.91	32.60	6.65	32.72	7.564
BMI (kg/m^2^)	17.91	0.7313	17.75	1.10	18.01	1.21	18.61	2.08	18.34	0.6198	18.13	1.197

BMI, body mass index; PG, physical guidance; SD, standard deviation.

**Table 2:** Descriptive to the error values obtained in three consecutive trials.

Tests	Groups of PG										Totally (*n* = 5)	
	0% (*n* = 5)		25% (*n* = 5)		50% (*n* = 5)		75% (*n* = 5)		100% (*n* = 5)			
	Mean	SD	Mean	SD	Mean	Sd	Mean	SD	Mean	SD	Mean	SD
Pre-test	17.68	0.54	17.88	0.71	17.51	0.65	18.26	0.45	18.03	0.85	17.87	0.65
Acquisition	7.80	0.76	8.61	1.38	5.10	0.71	3.27	-0.73	4.18	0.76	5.79	2.27
Retention	11.11	1.38	11.71	1.79	9.71	1.16	8.45	1.12	11.93	1.05	10.58	1.81

PG, physical guidance; SD, standard deviation.

**Table 3:** Multiple comparisons according to Tukey post-hoc tests.

Group (I)	Comparative Group (J)	Pre-Test		Acquisition		Retention	
		MD (I–J)	*p*	MD (I–J)	*p*	MD (I–J)	*p*
0%	25%	−0.20	0.99	−0.82	0.57	−0.60	0.95
	50%	0.17	0.99	2.70	≤0.001 *	1.40	0.48
	75%	−0.58	0.63	4.55	≤0.001 *	2.67	0.03 *
	100%	−0.35	0.91	3.62	≤0.001 *	−0.83	0.86
25%	50%	0.37	0.90	3.52	≤0.001 *	2.00	0.16
	75%	−0.38	0.88	5.37	≤0.001 *	3.27	0.007 *
	100%	−0.15	0.99	4.34	≤0.001 *	−0.22	<0.999
50%	75%	−0.75	0.39	1.85	0.03 *	1.27	0.57
	100%	−0.52	0.72	0.92	0.51	−2.22	<0.999
75%	100%	0.23	0.98	−0.93	0.50	−3.48	0.004 *

MD—mean difference; * represents significance at the level of *p* < 0.05.

## Data Availability

The datasets used and/or analyzed during the current study are available from the corresponding author on reasonable request.
